# Principles of Human Pathology, with Their Principal Application to Psychology, Therapeutics, Hygiene, and Forensic Medicine

**Published:** 1853-01

**Authors:** 


*Principles of Human Physiology* with their principal applications to Psychology, Therapeutics, Hygiene, and Forensic Medicine, by WILLIAM CARPENTER, M.D., F.R.S., F.G.S., examiner in Physiology and Comparative Anatomy in the University of London, &c. Fifth American from the fourth and enlarged London edition, with three hundred and fourteen illustrations. Edited with notes and additions, by FRANCIS GURNEY SMITH, M.D., Professor of the Institutes of Medicine in the Medical Department of Pennsylvania College; lecturer on Physiology in the Philadelphia Association for Medical Instruction, &c. Philadelphia; Blanchard & Lea, 1853. pp. 1091.

No branch of the science of Medicine has made more rapid progress than Physiology, during the last few years; hence the necessity for a complete remodelling of such as had been standard works upon this subject, or new and more recent productions would take their places.

Dr. Carpenter seems determined to retain his position, as one of the leading physiologists of the age; if industry and perseverance in the study of his favorite branch will continue to secure to him, as it has thus far, this enviable distinction. The new edition before us is so enlarged and improved in every respect, as to present as full and comprehensive a view of modern physiology as can be found in our language.

With this work by Dr. Carpenter from which to acquire knowledge of the principles of Physiology, and Todd and Bowman's Physiological Anatomy as the structural basis of such knowledge, no physician at the present day should allow himself to fall in the rear in the modern march in physiological knowledge.

For sale by A. H. & C. Burley, Chicago. H.

